# Efficacy, Safety, and Subject Satisfaction of PrabotulinumtoxinA for Moderate-to-Severe Crow’s Feet: A Phase IV, Multicenter, Double-Blind, Randomized, Placebo-Controlled Trial

**DOI:** 10.3390/jcm12196326

**Published:** 2023-10-01

**Authors:** Soo-Kyung Lee, Myoung Shin Kim, Soon-Hyo Kwon, Bo Young Chung, Se Hee Han, Hyoung Jun Kim

**Affiliations:** 1 Department of Dermatology, Inje University Sanggye Paik Hospital, Inje University School of Medicine, Seoul 01757, Republic of Korea; 2 Department of Dermatology, Kyung Hee University Hospital at Gangdong, Kyung Hee University School of Medicine, Seoul 05278, Republic of Korea; 3 Department of Dermatology, Hallym University Kangnam Sacred Heart Hospital, Hallym University College of Medicine, Seoul 07441, Republic of Korea; 4 Clinical Development Center, Daewoong Pharmaceutical Co., Ltd., Seoul 06170, Republic of Korea

**Keywords:** prabotulinumtoxinA, crow’s feet, patient satisfaction, facial wrinkle

## Abstract

PrabotulinumtoxinA has been identified as an effective agent against crow’s feet. Our study, which included Korean patients with moderate to severe crow’s feet, was undertaken to compare the efficacy and safety of PrabotulinumtoxinA and placebo treatments. Of the 90 study participants, 60 received prabotulinumtoxinA (24 U), whereas 30 received a placebo. The primary outcome assessment included facial wrinkle grading by investigators. At week 4, 69.64% of patients in the prabotulinumtoxinA group exhibited minimal crow’s feet severity; in contrast, a 0% improvement was observed in the placebo group (*p* < 0.0001). At week 12, the improvement rates were 30.36% for prabotulinumtoxinA and 6.90% for the placebo, demonstrating a significant difference (*p* = 0.0152). Based on the independent review panel’s assessment at week 4, the improvement rate was 39.29% in the prabotulinumtoxinA group and 3.45% in the placebo group during maximum smiling. Additionally, patient satisfaction was notably higher in the prabotulinumtoxinA group (32.14%) than in the placebo group (10.34%) at week 4 (*p* = 0.0289). Both treatments displayed comparable safety profiles, with only mild local reactions reported as ADRs for one patient from the prabotulinumtoxinA group. Thus, prabotulinumtoxinA demonstrates significant potential as a potent and safe remedy for crow’s feet.

## 1. Introduction

Crow’s feet, also known as periorbital wrinkles, are a prominent sign of aging that manifest as fine lines radiating from the corners of the eyes. These wrinkles, while not solely a cosmetic concern, can significantly affect individuals’ self-perception and social engagement. Primarily caused by the repetitive movement of the lateral orbicularis oculi and zygomaticus muscles [1], they become more noticeable with age and hence warrant effective treatment.

Within this context, botulinum toxin therapies have been increasingly recognized as minimally invasive yet effective modalities [2]. Notably, prabotulinumtoxinA has received approval from the Korean Ministry of Food and Drug Safety (MFDS) for the treatment of moderate-to-severe crow’s feet. This approval was informed by positive results from Phase II trials, where prabotulinumtoxinA demonstrated an improvement effect similar to that of onabotulinumtoxinA. Further validation came from Phase III split-face clinical trials, which not only confirmed its efficacy as comparable to that of onabotulinumtoxinA but also verified its safety profile for up to 16 weeks [3].

This study was undertaken to compare the efficacy and safety of prabotulinumtoxinA and placebo treatments for moderate-to-severe crow’s feet by analyzing the various evaluations conducted by investigators, an independent review panel (IRP), and patients.

## 2. Materials and Methods

### 2.1. Study Design and Ethics

A Phase IV clinical trial was executed across multiple sites in a randomized, double-blinded, and placebo-controlled setting from January to May 2022. This research was conducted in three distinguished medical facilities located in Seoul, the Republic of Korea: Sanggye Paik Hospital, Kyung Hee University Hospital at Gangdong, and Hallym University Kangnam Sacred Heart Hospital. Prior to the study’s commencement, the respective institutional boards or ethical committees of each facility granted their approvals (Approval code: SGPAIK-2021-10-013, KHNMC 2021-10-013, and HKS 2021-10-015). All participants provided written consent before participating in the study.

### 2.2. Patients

The inclusion criteria encompassed individuals aged between 19 and 65 years exhibiting moderate-to-severe crow’s feet during maximum smiling as determined by investigators’ assessments. An age range of 19 to 65 years was set for the study participants in accordance with the updated guidelines from the Korean Ministry of Food and Drug Safety (MFDS), which revised the legal age of adulthood to 19 years (from 18 years) in 2021. The exclusion criteria encompassed individuals with past negative experiences with botulinum neuromodulators; a history of neuromuscular diseases such as myasthenia gravis, Lambert–Eaton myasthenic syndrome, amyotrophic lateral sclerosis, or motor neuropathy; those who had received a botulinum toxin injection within the past 3 months or had undergone aesthetic interventions related to crow’s feet, such as surgeries, laser treatments, or periocular area augmentations within a year before evaluation; individuals with skin irregularities such as diseases, infections, or scars in the injection region; and those who were potentially pregnant or currently nursing.

### 2.3. Study Medication and Procedures

The investigators and patients were blinded to the types of drugs assigned. At every clinical trial location, participants were divided into either the prabotulinumtoxinA or placebo group using a 2:1 randomization method. Each vial of prabotulinumtoxinA contained 100 U of *Clostridium botulinum* toxin Type A and was mixed with 2.5 mL of 0.9% sterile saline immediately before administration. In contrast, the placebo group received 0.9% sterile normal saline. An independent professional ensured blinding by preparing the solutions and filling two identical syringes in line with patient code assignments. Using a fine 30- to 33-gauge needle, each patient received 0.1 mL (4 U) injections of prabotulinumtoxinA at three designated spots on each side of the lateral orbicularis oculi muscle, totaling 24 U. Those in the control group were treated similarly but with normal saline instead of prabotulinumtoxinA.

### 2.4. Efficacy Outcome Assessments

The efficacy and safety assessments were conducted at 4 and 12 weeks. Using a facial wrinkle scale (FWS) that ranged from 0 (none) to 3 (severe), the intensity of crow’s feet on each side of the face was gauged at both rest and maximum smiling [4]. For every assessment point, two evaluations were conducted for all participants: one in real-time by the investigators and another via standardized photos examined by three independent dermatologists. These dermatologists had no affiliations with the trial institutions and remained unaware of the treatment category and the point in the study timeline. A stringent protocol was followed when capturing photographs, ensuring consistent conditions in relation to parameters such as lighting, camera equipment, and settings. Both the investigators and the members of the IRP underwent training on applying the scale and were provided with a photographic atlas. This atlas, showcasing various severity grades, aimed to reduce grading inconsistencies.

Patient satisfaction regarding the improvement and overall appearance of crow’s feet was measured. A subjective global scale was used to gauge the change in crow’s feet severity from its initial state, offering a range of nine increments, from +4 (indicating a 100% improvement) to −4 (indicating a 100% deterioration). The degree of patient contentment with crow’s feet treatment and overall appearance was gauged across a seven-point scale, where grade 1 denoted “highly unsatisfied” and grade 7 signified “highly satisfied”. At each appointment, patients provided an estimation of their perceived age. Furthermore, the average shift in this perceived age was calculated, and the percentage of patients who believed they appeared more youthful than at the beginning of the study was determined.

The primary efficacy outcome was defined as the percentage of participants who, at the 4-week mark and during maximum smiling, were rated by the investigators as exhibiting either non-existent (grade 0) or mild (grade 1) crow’s feet. The secondary efficacy outcomes spanned various aspects: (a) the fraction of patients with crow’s feet rated as grade 0 or 1 during maximum smiling at week 12; (b) those who showed at least a 1-grade improvement from the baseline at rest at weeks 4 and 12; (c) the crow’s feet improvement rate during maximum smile and rest at weeks 4 and 12 as evaluated by IRP; (d) those who exhibited a crow’s feet improvement grade of +2 (reflecting a 50% enhancement) or more on the subjective global scale at weeks 4 and 12; (e) participants who rated their satisfaction with the treatment and appearance as high (6 or 7) at weeks 4 and 12; and (f) individuals who perceived themselves as looking more youthful at weeks 4 and 12.

### 2.5. Safety Assessments

Throughout the study, we diligently tracked any adverse events (AEs) and categorized them based on both their intensity and their association with the injection. Following the treatment, each patient was subjected to a 30 min observation period to detect immediate AEs. Every appointment involved a review of vital signs. Furthermore, at the commencement and conclusion of the study, laboratory tests, including pregnancy tests for potentially fertile women, and comprehensive physical check-ups were conducted.

Any AEs that manifested or intensified post-treatment were labeled as treatment-emergent adverse events (TEAEs), encompassing new AEs that emerge after drug administration and any escalation in the severity of a pre-existing condition relative to its initial state. An adverse drug reaction (ADR) is an unintended, harmful reaction to a drug and denotes a situation where a causal relationship with a drug cannot be ruled out [5].

### 2.6. Statistical Analysis

The complete analysis group, denoted as the full analysis set (FAS), comprised individuals who received the study drug and underwent at least one efficacy evaluation. The per-protocol set (PPS) was defined as patients who successfully completed the study without any protocol deviations. For safety assessments, we considered a subset of participants who had been administered the study drug at least once. Using the Cochran–Mantel–Haenszel method, in which the clinical trial data were adjusted for stratification factors (SAS 9.4; SAS Institute, Cary, NC, USA), the common risk difference between the two administration groups (test and control groups), 95% two-sided confidence interval, and *p*-values were determined; *p*-values < 0.05 were considered statistically significant. Cohen’s kappa was used to analyze the degree of agreement between the evaluations of the investigators and those of the IRP, and the results for each of the left and right sides and both sides were explored.

## 3. Results

### 3.1. Patients

**Figure 1:**
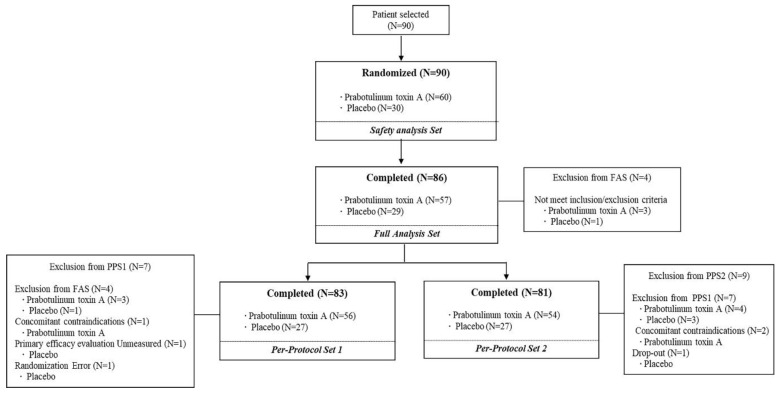
Flowchart detailing the inclusion of the study subjects.

Figure 1 depicts a flowchart of the study. All 90 screened patients were randomly assigned, with 60 in the prabotulinumtoxinA group and 30 in the placebo group. The efficacy outcomes of the FAS and PPS1 populations, which contained 86 and 83 patients, respectively, were evaluated at week 4, whereas the outcome of the PPS2 population, which included 81 patients who completed the study, was evaluated at week 12. 

Within the FAS cohort, patient ages spanned from 33 to 64 years. The average ages in the prabotulinumtoxinA and placebo groups were 50.51 (±8.33) years and 48.66 (±6.8) years, respectively. Of these patients, 77 were female (89.5%), whereas 9 were male (10.5%) (Table 1). The initial evaluations revealed that the participants had either moderate or severe crow’s feet during maximum smiling, with 36% and 64% of cases categorized as moderate and severe, respectively.

### 3.2. Efficacy Outcome Assessments

#### 3.2.1. Primary Outcome Assessments

**Figure 2:**
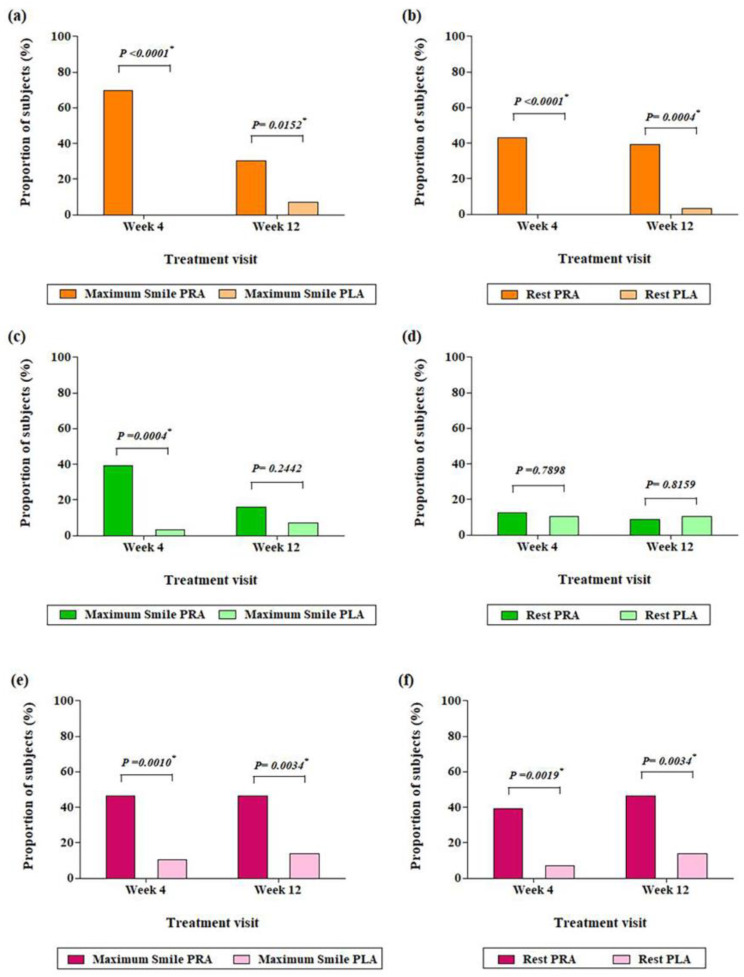
Proportion of subjects with (**a**) grade 0 or 1 crow’s feet severity during maximum smiling and (**b**) at least 1-grade improvement in crow’s feet severity at rest as evaluated by the investigators. Proportion of subjects with (**c**) grade 0 or 1 crow’s feet severity during maximum smiling and (**d**) at least 1-grade improvement in crow’s feet severity at rest as evaluated by the independent review panel. Proportion of subjects with grade +2 (50% improvement) or more improvement in crow’s feet severity (**e**) during maximum smiling and (**f**) at rest as evaluated by subjects. PRA = prabotulinumtoxinA; PLA = placebo. * Statistically significant at the two-sided significance level of 5%.

**Figure 3:**
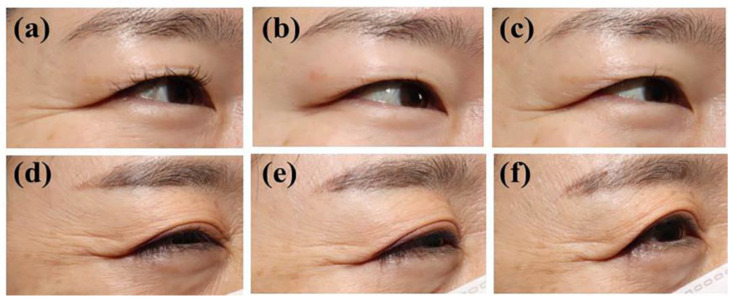
Representative photographs of crow’s feet during maximum smiling in a subject administered an prabotulinumtoxinA injection at (**a**) baseline, (**b**) week 4, and (**c**) week 12 and a placebo injection at (**d**) baseline, (**e**) week 4, and (**f**) week 12.

The final efficacy outcome assessment was performed on the FAS population. According to the evaluation of the investigators at week 4, the proportion of patients with improved crow’s feet during maximum smiling was 69.64% (39/56 patients) in the prabotulinumtoxinA group and 0% (0/29 patients) in the placebo group (Figure 2a), indicating a statistically significant difference (69.64%) (*p* < 0.0001) between groups. Photographic evidence showed an improvement in crow’s feet severity in each group (Figure 3).

#### 3.2.2. Secondary Outcome Assessments

- **Results of the Investigators’ Evaluation**

At week 12, the improvement rates of crow’s feet during maximum smiling were 30.36% (17/56 patients) and 6.90% (2/29 patients) in the prabotulinumtoxinA and placebo groups, respectively, indicating a statistically significant difference between both groups (*p* = 0.0152).

At week 4, the rates of patients with improved crow’s feet at rest were 42.86% and 0% in the prabotulinumtoxinA and placebo groups, respectively; however, at week 12, these figures changed to 39.29% and 3.45%, respectively (Figure 2b). Statistical differences in the rates of improvement were observed between both groups at both week 4 (*p* < 0.0001) and week 12 (*p* = 0.0004).

- **Results of the IRP’s Evaluation**

The crow’s feet improvement rates during maximum smiling in the prabotulinumtoxinA and placebo groups were 39.29% and 3.45%, respectively, at week 4 and 16.07% and 6.9%, respectively, at week 12. A statistically significant difference was observed between the two groups at week 4 but not at week 12 (week 4, *p* = 0.0004; week 12, *p* = 0.2442) (Figure 2c).

At week 4, the rates of patients with improved crow’s feet at rest were 12.5% and 10.34% in the prabotulinumtoxinA and placebo groups, respectively; however, at week 12, these rates changed to 8.93% and 10.34%, respectively. The differences between the groups were not statistically significant at either week 4 (*p* = 0.7898) or week 12 (*p* = 0.8159) (Figure 2d).

- **Patients’ Self-Assessment Results**

Crow’s feet improvement rates during maximum smiling in the prabotulinumtoxinA and placebo groups were 46.43% and 10.34%, respectively, at week 4 and 46.43% and 13.79%, respectively, at week 12. A statistically significant difference was observed between both groups at both time points (week 4, *p* = 0.001; week 12, *p* = 0.0034) (Figure 2e).

The crow’s feet improvement rates at rest in the prabotulinumtoxinA and placebo groups were 39.29% and 6.9%, respectively, at week 4 (*p* = 0.0019) and 46.43% and 13.79%, respectively, at week 12 (*p* = 0.0034) (Figure 2f).

During the assessment of satisfaction with the crow’s feet treatment, 46.43% of patients in the prabotulinumtoxinA group and 6.9% in the placebo group reported high satisfaction (grades 6 or 7) at the 4-week mark; at week 12, these figures were 37.5% in the prabotulinumtoxinA group but remained at 6.9% in the placebo group. The differences in satisfaction between both groups were statistically significant at the 4-week (*p* = 0.0003) and 12-week (*p* = 0.0031) marks (Table 2).

During the patients’ assessment of satisfaction with their appearance, the satisfaction rates in the prabotulinumtoxinA and placebo groups were 32.14% and 10.34%, respectively, at week 4 and 28.57% and 6.90%, respectively, at week 12. A statistically significant difference was observed between both groups at both time points (week 4, *p* = 0.0289; week 12, *p* = 0.0227).

The proportions of patients who felt they looked younger after treatment were 35.71% and 31.03% in the prabotulinumtoxinA and placebo groups, respectively, at week 4 and 32.14% and 20.69%, respectively, at week 12. No statistically significant differences were observed between both groups at any time point (week 4, *p* = 0.6886; week 12, *p* = 0.2691).

### 3.3. Safety Analysis

TEAEs were identified in 58 cases for 29 patients (48.33%, 29/60 patients) in the prabotulinumtoxinA group and 41 cases for 16 patients (53.33%, 16/30 patients) in the placebo group. Among them, two ADRs, in which a causal relationship with the drug could not be ruled out, occurred for one patient (1.67%, 1/60 patients) in the prabotulinumtoxinA group. The ADRs were mild local reactions at the injection site in the lateral canthus. One serious adverse event (SAE) occurred for one patient (1.67%, 1/60) in the prabotulinumtoxinA group, and deep vein thrombosis occurred in the left leg of a 43-year-old male; however, no causal relationship with the investigated drug was observed. Furthermore, no unexpected AEs or acute AEs were observed within 30 min of drug administration, and none of the AEs caused a dropout. No clinically significant abnormalities were observed in vital signs, physical examination results, or other safety-related examination results.

## 4. Discussion

Our study holds importance as it directly compares the efficacy and safety of prabotulinumtoxinA with a placebo among Korean adults with moderate to severe crow’s feet. The most compelling evidence was the significant difference in the proportion of patients who reached a ‘none/mild’ severity level at the 4-week milestone during maximum smiling: 69.64% in the prabotulinumtoxinA group versus 0.00% in the placebo group. (*p* < 0.0001) These findings are not only robust but also highly consistent with previous research on other botulinumtoxinA products, including onabotulinumtoxin at 66.7% and prabotulinumtoxin at 65.02% [3,6]. Furthermore, the therapeutic effectiveness of botulinumtoxinA in treating crow’s feet is well-documented. A comprehensive meta-analysis carried out in 2021 revealed that the success rates at the 4-week evaluation point were 12.4-fold, 6.4-fold, and 32.5-fold higher for onabotulinumtoxin (24 U), abobotulinumtoxin (30U), and incobotulinumtoxin (24U), respectively, when compared to a placebo [7]. Furthermore, when assessing the treatment success of prabotulinumtoxin (24U) in relation to onabotulinumtoxin (24U), a meta-analysis demonstrated equivalent efficacy between the two treatments, showing a 1.0-fold increase at 4 weeks and a 1.1-fold increase at 12 weeks [7].

Both the investigators and the IRP reported significant crow’s feet improvement rates during maximum smiling at week 4 in the prabotulinumtoxinA group; however, only the investigators reported a significant crow’s feet improvement rate at rest at week 4. A significant level of concordance was observed between the evaluations of the investigators and those of the IRP during maximum smiling (*p* < 0.001); however, the concordance was ˂0.6 at rest. This discrepancy may be due to the difference in the evaluation methods, as the investigators evaluated the patients in real time (“live”), whereas the IRP relied only on photographs. A study on the evaluation of nasolabial fold wrinkles also indicated differences between the two-dimensional photograph evaluation of an IRP and the three-dimensional (depth) visual evaluation of the investigators [8], which were attributed to the fact that detailed wrinkle depths could not be observed using only photographs. Given that botulinum toxin A achieves its aesthetic results by reducing muscle contractions associated with dynamic wrinkles, it is plausible that the most notable improvements in crow’s feet severity would be evident during maximum smiling rather than when the face is at rest [9]. Reinforcing this idea, earlier research on crow’s feet, which employed various botulinum toxin formulations, primarily focused on assessments during maximum smiling [3,10].

In aesthetic procedures, an important criterion for determining effectiveness was how well the expectations of the patients were met [11]. The self-assessment results of this study revealed that at weeks 4 and 12, the prabotulinumtoxinA group exhibited significantly higher satisfaction levels than the placebo group, and satisfaction with crow’s feet treatment exceeded 40% in the prabotulinumtoxinA group until week 12. Previous studies have also confirmed a treatment satisfaction rate of over 50% until week 12 [2], which was attributable to the advantages of botulinum toxin, such as rapid drug efficacy and minimal pain during the procedure. However, no statistically significant differences were observed between the groups regarding the proportion of patients who felt they looked younger at weeks 4 and 12. The perception of ‘looking younger’ is influenced by various factors, such as fine wrinkles, skin dryness, loss of elasticity, and the pigmentation of the entire face; therefore, careful interpretation is required.

Safety considerations are crucial when administering injections for crow’s feet. The known adverse effects of botulinum toxin A injections on the facial and ocular regions include local reactions, headaches, and eye ptosis, which can last several weeks [12]. A 2016 meta-analysis studying the adverse effects of botulinum toxin A in treating glabellar and crow’s feet lines found that there was no significant increase in side effects in the botulinum toxin A group compared to the control group, with the exception of a hematoma at the injection site (RR = 1.2) [13]. In our safety evaluation, ADRs were reported for just one patient in the prabotulinumtoxinA group, manifested as mild local reactions like pruritus and a rash around the injection site. These adverse events were expected and are common following botulinum toxin injections [14]. As for SAEs, one patient in the prabotulinumtoxinA group experienced deep vein thrombosis. However, this was deemed unrelated to the trial medication, as it was attributed to the patient’s pre-existing condition of dyslipidemia and the specific location of the thrombosis being in the left leg, far from the injection site. This study, consistent with previous research, further confirms the safety of prabotulinumtoxinA in the treatment of facial wrinkles.

This study had several limitations. First, the grading system for assessing severity was inherently subjective and contained potential ambiguities between close grades. Although raters underwent specialized training to reach an appropriate degree of agreement, achieving perfect concordance was not possible. Second, the sample was predominantly female and restricted to a Korean population, thereby introducing the potential for both gender and racial biases in the study outcomes. It is worth noting, however, that prabotulinumtoxin has been subjected to Phase 3 clinical trials that included diverse ethnic groups like Caucasians, African Americans, and Asians [15]. Moreover, in studies focused exclusively on men, prabotulinumtoxin has demonstrated comparable efficacy and side effect profiles to onabotulinumtoxin [16]. These considerations somewhat mitigate the limitations concerning gender and racial biases. Therefore, future studies should aim to include a more gender-balanced sample and a broader range of ethnic backgrounds to generalize the findings.

This study adopted strict methodologies. The differences between the assessments of the IRP and investigators emphasize the need for standardized criteria for assessing crow’s feet severity. The blinded reviews of the IRP likely contributed to greater consistency compared to the assessments made by individual clinicians. Despite these variations, there was a consistent agreement on efficacy, particularly when evaluated during maximum smiling.

## 5. Conclusions

In summary, the evaluations of the investigators, the IRP, and patients all indicate that prabotulinumtoxinA resulted in a significantly superior improvement compared to the placebo. This was observed in the treatment of moderate-to-severe crow’s feet among adults at the 4-week mark, specifically during maximum smiling, in a Korean population. Moreover, the patients reported increased levels of satisfaction and perceived improvement in appearance following the treatment with prabotulinumtoxinA. The safety of prabotulinumtoxinA was also validated, with no statistical differences observed in terms of AEs, ADRs, or SAEs between the two groups. This study has verified the safety and efficacy of prabotulinumtoxinA, and we consider it an effective and safe option for the improvement of crow’s feet among adults.

## Figures and Tables

**Table 1:** Baseline demographics and assessments (full analysis set).

	PrabotulinumtoxinA Group (N = 57)	Placebo Group (N = 29)
**Mean age (±SD), years**	50.51 (±8.33)	48.66 (±6.80)
**Sex, N (%)**		
Male	5 (8.77)	4 (13.79)
Female	52 (91.23)	25 (86.21)
**Investigator’s crow’s feet** **severity rating, N (%)**		
Moderate	22 (38.60)	9 (31.03)
Severe	35 (61.40)	20 (68.97)
**Subjects’ appearance satisfaction rating, n (%)**		
Grade 1	8 (14.04)	3 (10.34)
Grade 2	14 (24.56)	7 (21.14)
Grade 3	14 (24.56)	10 (34.38)
Grade 4	16 (28.07)	7 (24.14)
Grade 5	1 (1.75)	2 (6.90)
Grade 6	4 (7.02)	0 (0.00)
Grade 7	0 (0.00)	0 (0.00)

**Table 2:** Results of subjects’ satisfaction assessments (full analysis set).

	Week 4		Week 12	
	PrabotulinumtoxinA Group (N = 57)	Placebo Group (N = 29)	PrabotulinumtoxinA Group (N = 57)	Placebo Group (N = 29)
**Proportion of subjects satisfied with crow’s feet treatment**				
Number of subjects	56	29	56	29
Satisfied ^‡^, N (%)	26 (46.43)	2 (6.90)	21 (37.50)	2 (6.90)
Treatment difference	39.53		30.17	
[95% Confidence interval] ^†^, *p*-value	[22.91, 56.14], 0.0003 *		[14.25, 46.09], 0.0031 *	
**Proportion of subjects satisfied with appearance**				
Number of subjects	56	29	56	29
Satisfied ^‡^, N (%)	18 (32.14)	3 (10.34)	16 (28.57)	2 (6.90)
Treatment difference	21.94		21.13	
[95% Confidence interval] ^†^, *p*-value	[5.07, 38.80], 0.0289 *		[6.39, 35.87], 0.0227 *	
**Proportion of subjects convinced that they looked younger**				
Number of subjects	56	29	56	29
Younger ^§^, N (%)	20 (35.71)	9 (31.03)	18 (32.14)	6 (20.69)
Treatment difference	4.41		11.58	
[95% Confidence interval] ^†^, *p*-value	[–17.35, 26.16], 0.6886		[–7.77, 30.93], 0.2691	

* Statistically significant at the two-sided significance level of 5%. ^‡^ Definition of ‘Satisfied’: Grade at the time of evaluation was 6 (satisfied) or 7 (very satisfied); ^†^ Cochran–Mantel–Haenszel Method corrected by stratification factors for clinical trial institutions. ^§^ Definition of ‘looked younger’: Age at the time of evaluation by appearance < Age by appearance at screening (Visit 1).

## Data Availability

The data presented in this study are available on request from the corresponding author. The data are not publicly available due to privacy restrictions.
